# Clinically Suspected Fungal Laryngitis Masquerading as Laryngeal Carcinoma in a High-Risk Patient

**DOI:** 10.7759/cureus.110604

**Published:** 2026-06-10

**Authors:** Kushal Modi, Scott Gibbs, John Diks

**Affiliations:** 1 Ear, Nose, and Throat, Marshall University, Huntington, USA; 2 Otolaryngology, Marshall University, Huntington, USA; 3 Pathology, Marshall University Joan C. Edwards School of Medicine, Huntington, USA

**Keywords:** fungal infection, high-risk, laryngeal carcinoma, laryngitis, larynx

## Abstract

Fungal laryngitis is an uncommon clinical entity that may radiographically and symptomatically mimic laryngeal malignancy, posing diagnostic challenges. We present the case of a 62-year-old male with a 40-pack-year smoking history, inhaled corticosteroid use, hypertension, diabetes mellitus, hepatitis C, and substance and alcohol abuse who presented with progressive hoarseness, sore throat, and dyspnea. Computed tomography (CT) revealed a laryngeal mass causing supraglottic airway narrowing, initially suggestive of carcinoma. Fiberoptic laryngoscopy demonstrated extensive white patches of the oropharynx, hypopharynx, and larynx. The patient showed significant clinical improvement following intravenous fluconazole and steroid therapy. Subsequent microsuspension laryngoscopy with biopsy revealed acute/chronic mucositis with reactive epithelial changes but no malignancy. Special stains (Periodic acid-Schiff (PAS), Grocott's Methenamine Silver (GMS), and Acid-Fast Bacilli (AFB)) were negative for fungi, though prior antifungal response supported the diagnosis. This case underscores the importance of considering isolated fungal laryngitis in high-risk patients with laryngeal masses to avoid misdiagnosis and unnecessary interventions.

## Introduction

Fungal laryngitis is most commonly caused by *Candida albicans*, a fungus that exists in yeast, pseudohyphal, and hyphal forms [1]. It is a normal inhabitant of the oropharyngeal mucosae but could be notably pathogenic to immunocompromised individuals [2]. Diagnosis of fungal laryngitis often requires a high index of suspicion as symptoms and imaging present similarly to laryngeal carcinoma, gastrointestinal reflux disease (GERD), or granulomatous disease [2]. It is commonly diagnosed using Periodic Acid-Schiff (PAS) and Grocott's Methenamine Silver (GMS) staining and managed with oral nystatin in limited mucosal infection or systemic antifungal therapy such as fluconazole in more advanced cases [3]. 

Patients often experience hoarseness, dysphagia, pharyngalgia, dysphonia, and have evidence of abnormalities of the glottis or the entire larynx on laryngoscopy [4]. Many of the risk factors for fungal laryngitis exert their effects by impairing normal immune function and disrupting mucosal integrity [4]. Such factors include diabetes mellitus, chemotherapy, Acquired Immunodeficiency Syndrome (AIDS), smoking, radiotherapy, long-term antibiotic use, and inhaled corticosteroids [2, 4]. These conditions predispose patients to infection as opportunistic fungi exploit host responses through systemic and targeted physiological processes [4].

Laryngeal malignancies, notably squamous cell carcinoma, are of high suspicion in patients presenting with a mass originating arising from the upper aerodigestive tract [5]. They account for approximately one-third of head and neck cancers and are the most prevalent malignancy in otolaryngology [5]. These tumors are known to impact individuals across all demographics and can infiltrate multiple regions of the larynx, complicating the diagnosis and treatment approach [4]. Risk factors include smoking history, excessive alcohol use, and exposure to asbestos and industrial metal fumes [6]. Patients may present with dysphagia, hoarseness, persistent throat discomfort, unintended weight loss, airway obstruction, and referred ear pain [6]. Clinician awareness of laryngeal carcinoma presentation is warranted, as late-stage disease carries a poor prognosis and often requires a multimodal treatment strategy [6]. 

Isolated fungal laryngitis, though rare, can mimic carcinoma both clinically and radiographically, leading to diagnostic pitfalls [7, 8]. The overwhelming predisposition of opportunistic fungal infections in immunocompromised individuals, in conjunction with their similarities in presentation to laryngeal carcinoma, subjects them to frequent diagnostic confusion [7]. This may delay appropriate antifungal therapy and inappropriately prompt invasive procedures [7]. This report highlights a case of presumed fungal laryngitis clinically masquerading as laryngeal carcinoma in a high-risk patient, emphasizing the role of clinical context, potential diagnostic discordance, and treatment response in managing these suspected cases.

## Case presentation

A 62-year-old Caucasian male with hypertension, diabetes mellitus, hepatitis C, substance and alcohol abuse, inhaled corticosteroid use (budesonide), and a 40-pack-year smoking history presented to the emergency department with worsening hoarseness, odynophagia, and dyspnea over one week. Vital signs included tachycardia (heart rate (HR) 104 bpm), tachypnea (respiratory rate (RR) 23/min), and oxygen saturation of 95% on 3L nasal cannula. Auscultation revealed stridor and wheezing. Laboratory studies showed leukocytosis (WBC 13.9 k/μL) and mild anemia (hemoglobin (Hgb) 12.5 g/dL) (Table 1). CT neck with contrast identified a 2.8 × 2.0 × 3.1 cm midline laryngeal mass near the anterior commissure, causing supraglottic narrowing and left maxillary sinusitis (Figure 1). Fiberoptic laryngoscopy demonstrated extensive, confluent white plaques involving the oropharynx, hypopharynx, and larynx, highly suggestive of severe fungal infection.

**Table 1:** Initial Laboratory Evaluation *The Respiratory Pathogen Panel evaluated and confirmed negative for adenovirus, parainfluenza (types 1, 2, 3, 4), influenza A, influenza B, respiratory syncytial virus (RSV), human metapneumovirus, rhinovirus/enterovirus, seasonal coronaviruses (NL63, OC43, 229E, HKU1), *Mycoplasma pneumoniae*, *Chlamydia pneumoniae*, *Bordetella pertussis*, *Bordetella parapertussis*, and SARS-CoV-2.

Panel/Laboratory Parameter	Patient Result	Reference Range	Interpretation
HEMATOLOGY			
White Blood Cells (WBC)	13.9 k/cmm	4.5 - 11.0 k/cmm	High
Red Blood Cells (RBC)	5.68 m/cmm	4.30 - 5.90 m/cmm	Normal
Hemoglobin (Hgb)	12.5 gm/dL	13.5 - 17.5 gm/dL	Low
Hematocrit (Hct)	43.1%	41.0 - 50.0%	Normal
Platelets	373 k/cmm	150 - 450 k/cmm	Normal
Mean Corpuscular Volume (MCV)	75.9 fL	80.0 - 100.0 fL	Low
Mean Corpuscular Hemoglobin (MCH)	22 pg	27 - 33 pg	Low
Mean Corpuscular Hemoglobin Concentration (MCHC)	29 gm/dL	32 - 36 gm/dL	Low
Red Blood Cell Distribution Width - Coefficient of Variation (RDW-CV)	16.0%	11.0 - 15.0%	High
Red Cell Distribution Width - Standard Deviation (RDW-SD)	43.0 fL	39.0 - 46.0 fL	Normal
Mean Platelet Volume (MPV)	9.4 fL	9.0 - 12.2 fL	Normal
Segmented Neutrophils	74.0%	40.0 - 70.0%	Normal
Immature Granulocytes%	1.0%	0.0 - 0.5%	High
Lymphocytes%	14.9%	20.0 - 44.0%	Low
Monocytes%	8.6%	2.0 - 10.0%	Normal
Eosinophils%	1.1%	0.0 - 5.0%	Normal
Basophils%	0.4%	0.0 - 2.0%	Normal
Absolute Neutrophils	10.3 k/cmm	1.8 - 7.7 k/cmm	High
Absolute Immature Granulocytes	0.14 k/cmm	0.00 - 0.03 k/cmm	High
Absolute Lymphocytes	2.07 k/cmm	1.00 - 4.80 k/cmm	Normal
Absolute Monocytes	1.19 k/cmm	0.20 - 1.00 k/cmm	Normal
Absolute Eosinophils	0.15 k/cmm	0.00 - 0.50 k/cmm	Normal
Absolute Basophils	0.05 k/cmm	0.00 - 0.20 k/cmm	Normal
CHEMISTRY			
Potassium	4.0 mEq/L	3.5 - 5.1 mEq/L	Normal
Sodium	135 mEq/L	136 - 145 mEq/L	Low
Chloride	95 mEq/L	98 - 107 mEq/L	Low
Carbon Dioxide (CO_2_)	32 mEq/L	22 - 29 mEq/L	High
Anion Gap (AGAP)	8 mEq/L	3 - 11 mEq/L	Normal
Blood Urea Nitrogen (BUN)	16 mg/dL	7 - 20 mg/dL	Normal
Creatinine	0.79 mg/dL	0.60 - 1.20 mg/dL	Normal
Glucose	141 mg/dL	70 - 100 mg/dL	High
Calcium	10.1 mg/dL	8.6 - 10.3 mg/dL	Normal
Albumin	4.6 gm/dL	3.5 - 5.0 gm/dL	Normal
Total Protein	8.6 gm/dL	6.4 - 8.3 gm/dL	High
Alkaline Phosphatase (Alk Phos)	125 unit/L	44 - 121 unit/L	High
Aspartate Aminotransferase (AST)	8 unit/L	10 - 40 unit/L	Low
Alanine Aminotransferase (ALT)	14 unit/L	7 - 56 unit/L	Normal
Total Bilirubin	0.3 mg/dL	0.2 - 1.2 mg/dL	Normal
Estimated Glomerular Filtration Rate (Chronic Kidney Disease Epidemiology Collaboration) (eGFR (CKD-EPI))	100 ml/min/1.73m²	> 60 ml/min/1.73m²	Normal
Magnesium	1.9 mg/dL	1.7 - 2.2 mg/dL	Normal
IMMUNOLOGY/SEROLOGY			
Mononucleosis Screen	Negative	Negative	Normal
MICROBIOLOGY/MOLECULAR			
Respiratory Pathogen Panel (PCR)*	Not Detected	Not Detected	Normal
Rapid Strep Test	Negative	Negative	Normal

**Figure 1:**
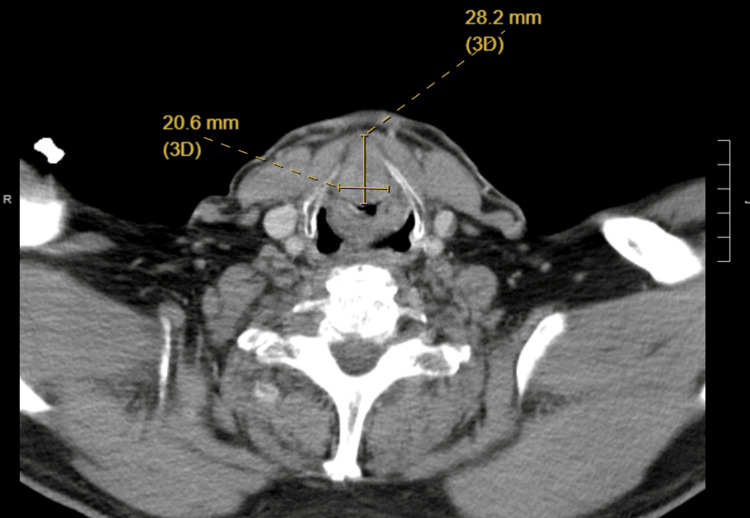
CT neck with contrast (axial view) demonstrating a midline laryngeal mass causing supraglottic narrowing.

The patient was admitted and treated with intravenous fluconazole (400 mg daily) and systemic steroids, resulting in a rapid and marked improvement in dyspnea and dysphagia, suggesting a multifactorial response to both antimicrobial and anti-inflammatory therapies. On admission, blood cultures and throat cultures were negative for fungal or bacterial growth, though a lower respiratory tract sample demonstrated rare fungal hyphae. Due to persistent clinical concern for a primary malignancy, microsuspension laryngoscopy with biopsy was performed. Intraoperatively, a 1 cm left vallecular cyst was excised, and a firm submucosal epiglottic mass was biopsied. Pathology revealed inflamed granulation tissue, chronic mucositis with reactive epithelial changes of the epiglottis, and necroinflammatory debris within the mass. Special stains (GMS, PAS, AFB) were negative for fungal organisms or acid-fast bacilli; however, the highly suggestive endoscopic appearance, the rare lower respiratory findings, and the robust clinical response supported a presumptive diagnosis of fungal infection (Figure 2). The patient was discharged on oral fluconazole with otolaryngology follow-up. During the post-discharge period, the patient returned to the outpatient clinic twice, presenting with similar but significantly less severe symptoms; both episodes were successfully managed with oral antifungals and a short course of steroids.

**Figure 2:**
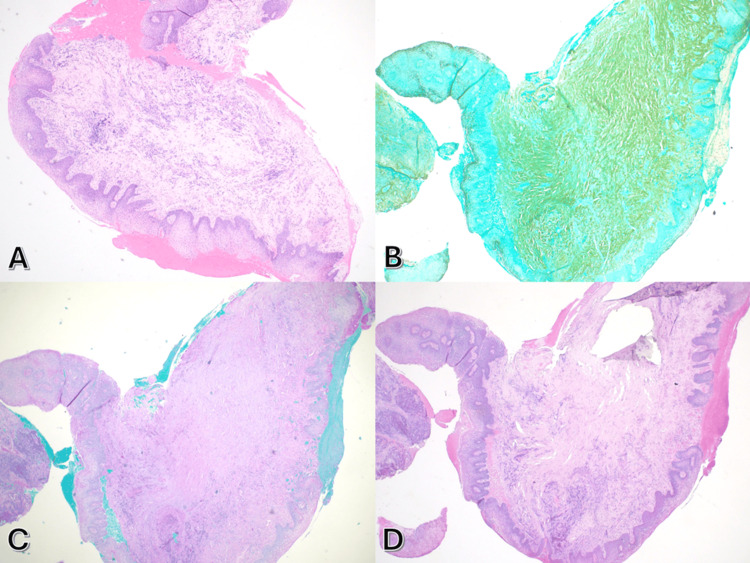
Histopathologic examination of the laryngeal biopsy. (A) Hematoxylin and Eosin (H&E) stain demonstrating squamous mucosa with underlying non-specific chronic inflammation; no definitive fungal organisms are identified. (B) Grocott’s Methenamine Silver (GMS) stain, (C) Acid-Fast Bacilli (AFB) stain, and (D) Periodic Acid–Schiff (PAS) stain, all of which were negative for fungal elements, pseudohyphae, or acid-fast bacilli within the subepithelial tissue. All panels are shown at low-power magnification (40x).

## Discussion

This case illustrates three key challenges: 1. the overlap in presentation between clinically suspected fungal laryngitis and malignancy, 2. risk stratification in conjunction with the limitations of histopathological identification, and 3. the rarity of fungal laryngitis itself.

Fungal laryngitis commonly manifests with throat pain, dysphonia, dysphagia, and hoarseness, with more advanced cases, like in the present case, also demonstrating stridor and respiratory distress [4]. These symptoms closely overlap with those of laryngeal carcinoma, with referred otalgia being one of the few features that, if present, would strongly suggest malignancy [6]. A hospital fiberoptic exam demonstrated extensive, confluent white plaques involving the oropharynx, hypopharynx, and larynx, while a laryngeal mass was identified on CT (Figure 1). Microsuspension laryngoscopy with biopsy was performed due to significant concern for malignancy and to definitive guide management. Endoscopic evaluation revealed a firm, submucosal midline epiglottic mass and a 1 cm left vallecular mucous retention cyst, while the biopsy demonstrated marked acute and chronic mucositis with fibrosis, mucinous/myxoid material, and reactive epithelial changes, with no fungal or mycobacterial organisms identified on special stains. A histopathological analysis confirming candidal laryngitis might include yeast-like features with pseudohyphae, spore-ridden necrotic tissue, and an overlying inflammatory exudate [7], whereas the majority of laryngeal cancers would display atypical squamous cells suggesting carcinoma [6-8].

It is important to note that inhaled steroids, diabetes mellitus, a relative immunocompromised state, and smoking were all present in this patient, creating a highly favorable milieu for candidal overgrowth [2, 4]. The patient received a short course of antibiotics for an ear infection a few months prior, which does not constitute the long-term antibiotic exposure typically considered a primary risk factor for fungal laryngitis [4]. Risk factor overlap often begins in the patient’s history, as laryngeal cancer tends to be more common in patients with prolonged tobacco use as well as excessive alcohol consumption [4]. Such a patient history often makes a cancer diagnosis the initial and statistically more likely consideration [5]. The mass on CT and symptoms like hoarseness and stridor initially strongly suggested carcinoma. Before imaging and biopsy, symptom overlap made distinguishing fungal laryngitis exceptionally difficult.

Beyond malignancy, the clinical presentation and negative histopathology require a broad differential diagnosis including non-infectious, environmental, and substance-induced etiologies. Given that this patient resides in a region with high environmental and industrial exposure, an environmental granuloma-such as an asbestos-induced granulomatous reaction-remains an important diagnostic consideration, as chronic particulate inhalation can induce localized mass-like lesions. Furthermore, the patient’s severe alcohol and substance abuse can independently disrupt mucosal barriers, impair ciliary clearance, and induce severe, non-specific chronic inflammation or foreign-body-type granulomas that radiographically and visually mimic neoplasia. Other granulomatous differentials, including sarcoidosis, atypical mycobacterial infections, or autoimmune granulomatosis with polyangiitis, must also be considered when standard biopsies are non-diagnostic. However, a triad of diabetes, hepatitis C, and inhaled corticosteroid use contributing to localized immunosuppression, coupled with the acute mucosal injury from smoking and alcohol, was a key factor supporting a presumed fungal etiology. These findings are also consistent with prior reports where fungal infections mimicked tumors [7-9]. Further, this phenomenon of fungal laryngitis mimicking laryngeal carcinoma is not limited to *Candida *infections, with similar presentations also being reported in aspergillosis cases [10, 11].

While risk factors help guide diagnosis, substantial challenges remain, particularly regarding the discordance between strong clinical suspicion and negative pathology. Mesolella et al. reported a case that highlights the association of this pathology with inhaled corticosteroid use, with many other studies also expressing this correlation [9, 12-14]. This detail may be overlooked when other diagnostics suggest carcinoma. Interestingly, the association between immunocompromised patients and laryngeal candidiasis, while well-documented and present in this case, is not a constant in the literature, as Nerukar et al. and others have seen immunocompetent cases in significant numbers [4, 8, 15]. This underscores the necessity of a holistic approach in identifying suspected fungal laryngitis.

Although biopsy remains the gold standard for definitive tissue confirmation, a positive response to therapy is a highly valuable diagnostic surrogate when pathology is inconclusive [3]. However, the rapid symptomatic and respiratory improvement observed during admission cannot be attributed solely to fluconazole; the concurrent administration of systemic corticosteroids likely played a confounding, significant role by rapidly reducing airway edema and localized laryngeal inflammation. Furthermore, the complete absence of fungal elements on PAS and GMS stains underscores prominent diagnostic limitations. Biopsies may yield false negatives due to superficial or non-representative tissue sampling, prior antifungal treatment, technical or staining artifacts in dense, necroinflammatory debris, or the presence of superficial colonization rather than deep tissue invasion, which can obscure fungal elements despite a high clinical fungal load [16].

The rarity of isolated candidal laryngitis poses an additional challenge in its prompt and accurate diagnosis [4, 8, 14]. The condition has been increasingly mentioned in literature over the last 30 years; however, only a limited number of cases are documented annually [4, 14]. In 2009, the most comprehensive case series at the time reported only 54 laryngeal candidiasis patients over a 10-year period [14]. A more recent four-year study (2016-2019), also conducted at a voice clinic, reported only 55 cases, which is notable given that such clinics frequently receive referrals for hoarseness, a key symptom of the condition [4]. The difficulty in accurately stratifying risk and navigating negative biopsy results is only underscored by the numerous case reports that document laryngeal candidiasis being initially misdiagnosed as laryngeal carcinoma.

## Conclusions

A diagnosis of fungal laryngitis warrants careful consideration in the differential diagnosis of laryngeal masses, particularly in individuals with localized or systemic immunosuppression. This is especially relevant for patients with a combination of predisposing risk factors, including the use of inhaled corticosteroids, a history of smoking, chronic substance abuse, or diabetes mellitus. Because radiographic and clinical features can closely mimic malignancy, an unrecognized fungal etiology may potentially lead to unnecessary, invasive surgical interventions. While definitive tissue confirmation remains ideal, empiric antifungal therapy may be considered in carefully selected high-risk patients when clinical and endoscopic suspicion remains high despite nondiagnostic pathology, as a multi-treatment clinical response can help support the presumptive diagnosis. Enhanced clinician awareness of these diagnostic pitfalls and early otolaryngology involvement are essential to mitigate diagnostic delays and optimize patient management.
